# Bcl-x_L_ antisense oligonucleotides radiosensitise colon cancer cells

**DOI:** 10.1038/sj.bjc.6601254

**Published:** 2003-09-30

**Authors:** V Wacheck, E Selzer, P Günsberg, T Lucas, H Meyer, C Thallinger, B P Monia, B Jansen

**Affiliations:** 1 Department of Clinical Pharmacology, Section of Experimental Oncology/Molecular Pharmacology, University of Vienna, Währinger Gürtel 18-20, A-1090 Vienna, Austria; 2 Department of Radiotherapy and Radiobiology, University of Vienna, Währinger Gürtel 18-20, A-1090 Vienna, Austria; 3 Isis Pharmaceuticals Inc., 2292 Faraday Avenue, Carlsbad, CA 92008, USA; 4 The Prostate Centre, University of British Columbia, 2733 Heather Street, Vancouver, BC, Canada V5Z 3J5

**Keywords:** antisense oligonucleotides, Bcl-x_L_, radiosensitisation, colon cancer

## Abstract

Advanced colon cancer is a malignancy with poor response to various treatment modalities including ionising radiation (IR) and chemotherapy. Both IR and chemotherapeutic agents have been shown to act by inducing apoptosis, a type of cell death antagonised by the Bcl-x_L_ gene product. Since approximately 60% of human colon cancers express Bcl-x_L_, it was the aim of this study to explore the potential of Bcl-x_L_ antisense oligonucleotides as a novel radiosensitisation strategy. Caco-2 colon cancer cells were treated with Bcl-x_L_ antisense oligonucleotides in combination with IR or cisplatin, and Bcl-x_L_ protein expression, apoptosis, cell viability and clonogenic survival were examined. Bcl-x_L_ antisense oligonucleotide specifically reduced the Bcl-x_L_ protein level by almost 50% in Caco-2 cells. The decreased threshold for the induction of apoptosis resulted in a 300% increase of apoptosis after IR or cisplatin treatment and led to a 60% reduction of cell proliferation beyond response rates achieved with IR. These data suggest that Bcl-x_L_ is an important factor contributing to the treatment resistance of human colon cancer. Specific reduction of Bcl-x_L_ protein levels by antisense oligonucleotides qualifies as a promising therapeutic strategy for colon cancer that may help overcome resistance and improve clinical outcome in this malignancy.

Colorectal carcinoma is the second leading cause of cancer death in Western countries with an incidence rate of 1 : 3000 (Midgley and Kerr, 1999; Greenlee *et al*, 2000). Surgical resection is the first choice of therapy for localised tumours, but at least 40% of patients with colorectal cancer will develop local recurrence or metastases during the course of the disease. For patients with advanced colorectal cancer, adjuvant chemotherapy and/or ionising radiation (IR) offer a small but significant survival advantage (Midgley and Kerr, 1999; Wils *et al*, 2001). While in the US postoperative (chemo)radiotherapy is considered the adjuvant treatment of choice, most European investigators have advocated for preoperative intensive short-course irradiation instead (Wils *et al*, 2001). Nevertheless, irrespective of the therapeutic strategy selected, advanced colorectal cancer remains a prime example for poor response to adjuvant treatment due to low sensitivity to both IR and chemotherapy.

The mechanisms responsible for the resistance of this malignancy to IR or chemotherapeutic drugs are not yet fully understood. Apoptosis is currently a subject of intense research, and there is growing evidence that tumour cells, at least in part, die by apoptosis in response to IR or cytotoxic treatments (Desoize, 1994; Huang *et al*, 1997; Coultas and Strasser, 2000; Evan and Vousden, 2001; Reed, 2001). The members of the Bcl-2 multigene family are a pivotal set of apoptotic regulators that consist of partially interacting proteins highly conserved from nematodes to mammals (Kroemer, 1997; Antonsson and Martinou, 2000; Tsujimoto and Shimizu, 2000). Among the various Bcl-like proteins, the effects and functions of Bcl-x in controlling apoptosis induced by IR or chemotherapy have been studied recently. The Bcl-x gene is a Bcl-2 homologue and plays an important role in the regulation of programmed cell death in a variety of tissues (Xerri *et al*, 1998; Tsujimoto and Shimizu, 2000). Bcl-x is alternatively spliced into two mRNAs. The protein product of the larger Bcl-x mRNA (Bcl-x_L_) functions as a repressor of programmed cell death (Kroemer, 1997), whereas the smaller splicing product Bcl-x_S_, encodes a protein capable of accelerating cell death (Antonsson and Martinou, 2000; Tsujimoto and Shimizu, 2000). While it becomes increasingly clear that the two close relatives Bcl-2 and Bcl-x_L_ show different cellular expression patterns and may complement each other's antiapoptotic function, the exact mechanisms of action remain unclear (Kroemer and Reed, 2000; Robertson and Orrenius, 2000).

The antiapoptotic effects of Bcl-x_L_ against IR- and chemotherapy-induced apoptosis have been demonstrated in various human cancer cell lines (Huang *et al*, 1997; Amarante-Mendes *et al*, 1998; Nagane *et al*, 1998; Srinivasan *et al*, 1998). The most pronounced effects were observed in cells containing the highest levels of Bcl-x_L_ expression.

Antisense (AS) oligonucleotides are modified single-strand stretches of nucleotides capable of inhibiting protein expression by complexing with the complementary target mRNA preventing translation. Antisense oligonucleotides hold great promise as agents for specific manipulation of gene expression and have been used to inhibit gene expression both *in vitro* and *in vivo* (Kitada *et al*, 1994; Keith *et al*, 1995). Bcl-x_L_ downregulation by AS oligonucleotides has been observed in different types of cancer cells leading to an increase in susceptibility to apoptotic stimuli (Amarante-Mendes *et al*, 1998; Lebedeva *et al*, 2000). Recently, it was shown that Bcl-x_L_ AS oligonucleotides are capable of sensitising colon cancer cells *in vitro* to 5-fluorouracil (Nita *et al*, 2000). Furthermore, bcl-2/bcl-x_L_ bispecific oligonucleotides significantly reduced Bcl-x_L_ expression that leads to increased apoptosis and delayed tumour growth in a xenotransplantation model for colon cancer (Gautschi *et al*, 2001). Taylor *et al* (1999) demonstrated specific downregulation of Bcl-x_L_ by AS oligonucleotides (ISIS 16009) in keratinocytes and epithelial cells and sensitisation to UV-B radiation- and cisplatin-induced apoptosis. However, the effect of Bcl-x_L_ AS oligonucleotides on radiosensitivity of colon cancer has not yet been explored.

Given the overexpression of Bcl-x_L_ protein in more than 60% of human colon cancers (Krajewska *et al*, 1996; Maurer *et al*, 1998) and its positive correlation with poor prognosis (Biroccio *et al*, 2001), we hypothesised that downregulation by Bcl-x_L_ by AS oligonucleotides may sensitise colon cancer cells to IR or cisplatin.

## MATERIALS AND METHODS

### Cell culture

The human colorectal carcinoma cell line Caco-2 was obtained from American Type Culture Collection (ATCC, Manassas, VA, USA) and maintained in basal tissue culture medium (DMEM) supplemented with 8% foetal calf serum, 1% penicillin, and 1% streptomycin (all Gibco BRL, Paisley, UK) in a humidified 5% CO_2_, 95% ambient air atmosphere at 37°C. For treatment, Caco-2 cells were incubated with oligonucleotides and exposed to IR or cisplatin at the time points and concentrations as indicated. Cells were irradiated with a conventional radiation source (Stapilipan, Siemens, Munich, Germany) at a dose rate of 1 Gy min^−1^. Cisplatin was obtained from Ebewe (Unterach, Austria).

### Immune blotting

Western blotting of lysed oligonucleotide-treated cells was performed using chemiluminescence detection (Tropix, Bedford, MA, USA). Antibodies reacting with Bcl-x and actin were obtained from BD PharMingen (Franklin Lakes, NJ, USA) and Sigma (St Louis, MO, USA), respectively. Equal protein loading in each lane was documented by actin protein expression. The expression levels of proteins were determined by densitometric analysis of autoradiogramms with a Herolab E.A.S.Y. RH densitometer (Herolab, Wiesloch, Germany) and the E.A.S.Y. Win32 software (Herolab). Signal strength of each Bcl-x signal was normalised to actin and the ratios between Bcl-x protein expression in AS oligonucleotides-treated cell extracts and control extracts were calculated. Changes of protein expression below 20% were not regarded as significant.

### Oligonucleotides

HPLC purified 20-mer 2′-*O*-methoxyethyl chimerical phosphorothioate oligonucleotides complementary to the human Bcl-x_L_ were provided by ISIS Pharmaceuticals (Carlsbad, CA, USA). The sequence of the Bcl-x_L_ AS oligonucleotide ISIS 16009 was 5′-CTA CGC TTT CCA CGC ACA GT-3′. An 8-base mismatch (MM) oligonucleotide (ISIS 16967) 5′-CTC CAA TGT CCC CTC AAG GT-3′ was used as an internal control oligonucleotide. Underlined bases indicate 2′-*O*-methoxyethyl modification. For the screening experiments, further Bcl-x_L_ antisense oligonucleotides were tested: ISIS 15999 (5′-TCC CGG TTG CTC TGA GAC AT-3′), ISIS 16011 (5′-CTG GAT CCA AGG CTC TAG GT-3′), and ISIS 22783 (5′-CTG GAT CCA AGG CTC TAG GT-3′). All oligonucleotides were resuspended in 0.9% saline solution.

### Delivery of oligonucleotides

Cells were seeded at a density of 0.25 × 10^6^ ml^−1^ in six-well plates 24 h before oligonucleotide treatment. Cultures were then incubated for 4 h at 37°C with 200 nM oligonucleotide in the presence of 10 *μ*g ml^−1^ lipofectin (Gibco) as an uptake enhancer, according to the manufacturer's protocol. After incubation, the oligonucleotide–lipofectin mixture was replaced by complete medium and cells were cultivated as described above. For the screening experiments, cells were incubated for 48 or 72 h with oligonucleotides at a concentration of 50 *μ*M without uptake-enhancing lipofectin.

### Assessment of cell viability and clonogenic survival

For the assessment of cell growth *in vitro*, cells were incubated with oligonucleotides and exposed to IR at the time points and doses as indicated. Cisplatin was used at a dose almost doubling the number of apoptotic cells compared to untreated cells (50 *μ*M). At 24, 48, 72, and 96 h after oligonucleotide treatment, the number of viable cells was determined by a tetrazolium salt-based assay (WST-1 assay, Roche Diagnostics, Basel, Switzerland).

For determination of clonogenic survival following IR, cells were seeded in six-well plates and exposed to increasing single doses of IR. Postirradiation cells were plated in 6 cm dishes at a seeding density of approx. 1000 cells per well (in triplicate). After an incubation period of 10 days, culture dishes were stained with crystal violet and colonies of >50 cells were counted at low magnification.

### Flow cytometry

Apoptotic cells were identified by their sub-diploid DNA content using flow cytometrical analysis as previously described (Nicoletti *et al*, 1991). Cells were washed in PBS, fixed in ice-cold 70% ethanol for a minimum of 1 h, washed in PBS and incubated in PBS containing 0.1% DNase-free RNase A and 100 *μ*g ml^−1^ propidium iodide for 30 min and 1.5 × 10^4^ events analysed on a FACScalibur flow cytometer (Becton Dickinson, NJ, USA) with an argon laser tuned at 488 nm. Gates were set to exclude subcellular particles. The percent gated populations represent cells that are hypochromatic due to chromatin condensation and contain subdiploid DNA contents (percentage of apoptotic cells). The apoptotic morphology of this cell population was confirmed by fluorescence microscopy.

### Statistical analysis

Statistical significance between treatment groups was determined using one-way ANOVA and Bonferroni *post hoc* test analysis. *P*-values of <0.05 were considered to be of statistical significance.

## RESULTS

### Specific downregulation of Bcl-x_L_ in Caco-2 cells

**Figure 1:**
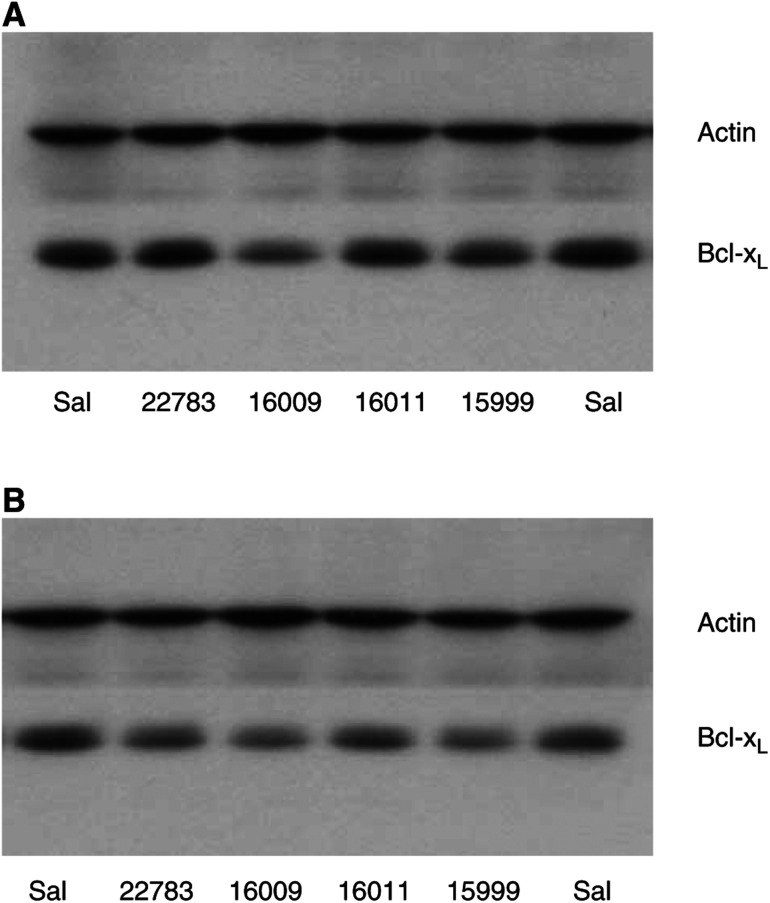
Screening of Bcl-x_L_ AS oligonucleotides: Western blots of Caco-2 cells 48 h (**A**) and 72 h (**B**) after treatment with four different AS oligonucleotides at a concentration of 50 *μ*M; lane 1: saline (Sal), lane 2: ISIS 22783, lane 3: ISIS 16009, lane 4: ISIS 16011, lane 5: ISIS 15999, and lane 6: saline (Sal).

In a screening experiment to identify the most potent Bcl-x_L_ AS oligonucleotides, Caco-2 cells were incubated with four different AS oligonucleotides targeting different sites of the Bcl-x mRNA as described in the Material and Methods section. After a 48-h incubation period at a concentration of 50 *μ*M, the Bcl-x_L_ AS oligonucleotides ISIS 16009 targeting the translation initiation codon site of Bcl-x_L_ resulted in the most prominent downregulation of Bcl-x_L_ protein expression by approximately one-third compared to the saline control (Figure 1A

). Although a longer incubation period of 72 h revealed marked downregulation of Bcl-x protein by all the AS oligonucleotides applied, ISIS 16009 was still the most potent AS oligonucleotide (Figure 1B).

**Figure 2:**
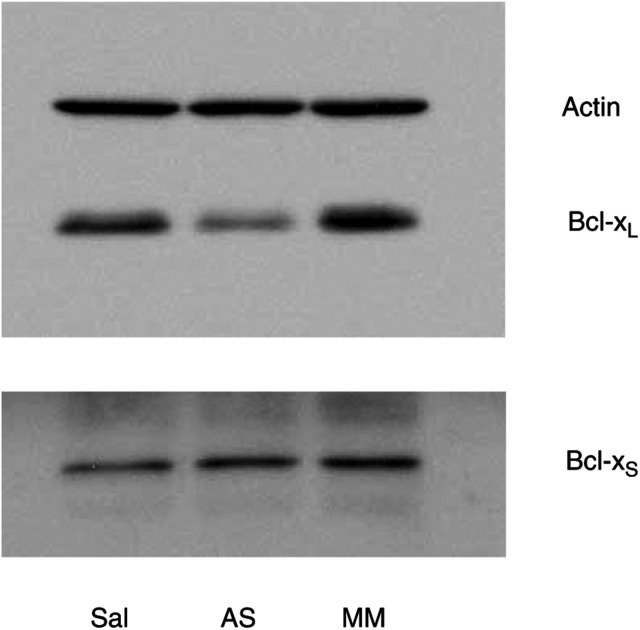
Bcl-x_L_ downregulation by Bcl-x_L_ AS oligonucleotides (ISIS 16009): Western blot of Caco-2 cells 48 h after a 4-h treatment with 200 nm oligonucleotides in the presence of 10 *μ*g ml^−1^ lipofectin; lane 1: saline (Sal), lane 2: ISIS 16009 Bcl-x_L_ AS oligonucleotides (AS), lane 3: 8-base mismatch oligonucleotides (MM). A representative blot of four independent experiments is presented.

We therefore focused further experiments on ISIS 16009 as the lead compound. Using an uptake-enhancing lipid (lipofectin), oligonucleotide concentrations were reduced to nanomolar concentrations minimising possible nonspecific oligonucleotide effects reported earlier at micromolar concentrations (Stein, 1995). Treatment of Caco-2 cells with 200 nm ISIS 16009 for 4 h in the presence of lipofectin led to a significant reduction (*P*<0.001) in Bcl-x_L_ expression after 48 h by almost 50% compared to saline control (Figure 2

; 47% AS/Sal, s.d. ±5%). No significant change of Bcl-x_L_ protein expression in cells incubated in the presence of the same concentration of MM oligonucleotide were observed (110% MM/Sal, s.d. ±9%; *P*>0.13). Concomitantly performed Western blot analysis of the cellular lysates demonstrated no changes in Bcl-x_S_ expression levels after oligonucleotide treatment (Figure 2; 96% AS/Sal, s.d. ±5%; 86% MM/Sal, s.d. ±4%; both *P*>0.1). Prolongation of the incubation period to 72 h led to no more pronounced downregulation of Bcl-x_L_ protein expression (data not shown).

### Bcl-x_L_ AS oligonucleotides lower the apoptotic threshold

**Figure 3:**
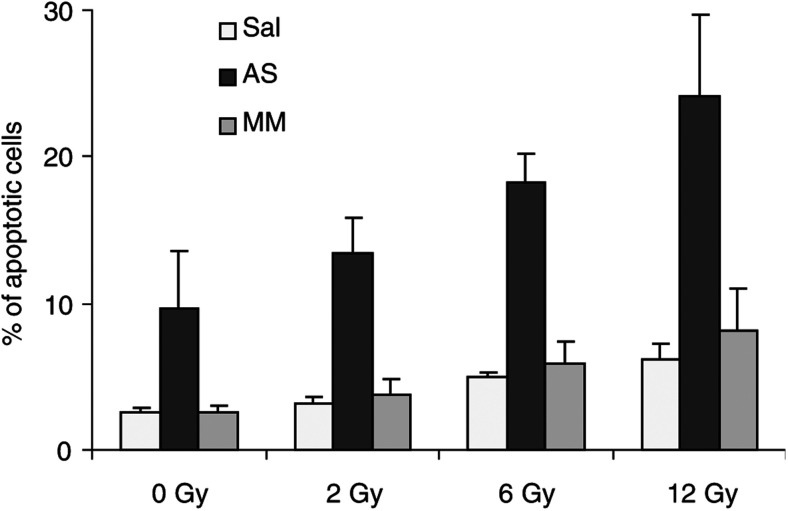
Bcl-x_L_ AS oligonucleotides facilitate the induction of apoptosis in human colon cancer cells. Caco-2 cancer cells were incubated for 4 h with saline (Sal), antisense (AS), or eight-base mismatch (MM) oligonucleotides at a concentration of 200 nM in the presence of 10 *μ*g ml^−1^ lipofectin. After 48 h, cells were treated with increasing doses of IR (0–12 Gy). At 96 h after oligonucleotide treatment, cells were harvested and analysed by FACS for apoptosis. Columns represent mean percentages of apoptotic cell death from four independent experiments; bars=s.d.

To study the influence of Bcl-x_L_ AS oligonucleotides on facilitating apoptosis in Caco-2 cells, the relative percentage of apoptotic cells compared to untreated controls was assessed by flow cytometry. Cells with a sub-G_0_/G_1_ DNA content due to chromatin condensation were considered apoptotic (Nicoletti *et al*, 1991). Caco-2 cancer cells were incubated for 4 h with saline, ISIS 16009 AS, or MM oligonucleotides at a dose of 200 nM in the presence of uptake-enhancing lipofectin. After a 48-h resting period, Caco-2 cells were treated with IR. Increasing doses of IR (0–12 Gy) resulted in a dose-dependent rise in the number of apoptotic cells up to a doubling of apoptotic cells at a dose of 12 Gy compared to nonirradiated cells. Treatment of Caco-2 colon cells with ISIS 16009 Bcl-x_L_ AS oligonucleotides alone significantly enhanced the rate of apoptotic cells compared to saline controls (Figure 3

; *P*<0.05), whereas no significant increase of apoptotic cell death in the group treated with MM oligonucleotides was observed. However, the combination of Bcl-x_L_ AS oligonucleotides and IR resulted in a pronounced increase of apoptotic cell death by about 300% compared to irradiated Caco-2 cells pretreated with either saline or MM oligonucleotides at all IR doses examined (Figure 3). These differences were highly statistically significant (*P*<0.001). The combination of ISIS 16009 Bcl-x_L_ AS oligonucleotide and an IR dose of 12 Gy approximately doubled the rate of apoptotic cells compared to AS oligonucleotide treatment alone (*P*<0.012). No statistically significant differences were observed between the saline control and MM oligonucleotide pretreated cells supporting a specific Bcl-x_L_ AS oligonucleotide mode of action.

Additionally, we investigated the combination of Bcl-x_L_ AS oligonucleotides and the chemotherapeutic agent cisplatin. Cisplatin at a dose of 50 *μ*M in combination with ISIS 16009 AS oligonucleotides almost doubled the rate of apoptotic cells compared to saline or MM oligonucleotides pretreated cells (Sal+Cis 7.2%, s.d. ±2.7%; AS+Cis 18.9%, s.d. ±5.9%; MM+Cis 5.1%, s.d. ±1.7%; both *P*<0.002; data not shown). Levels of apoptosis after cisplatin exposure in cultures pretreated with MM oligonucleotides did not differ significantly from the ones in the saline group.

### Bcl-x_L_ AS oligonucleotides radiosensitise Caco-2 cells

To determine the influence of Bcl-x_L_ AS oligonucleotides on cell viability and treatment resistance, Caco-2 colorectal cancer cells were treated with ISIS 16009 Bcl-x_L_ AS, MM oligonucleotides or saline in combination with IR at the same time points and concentrations as described above.

**Figure 4:**
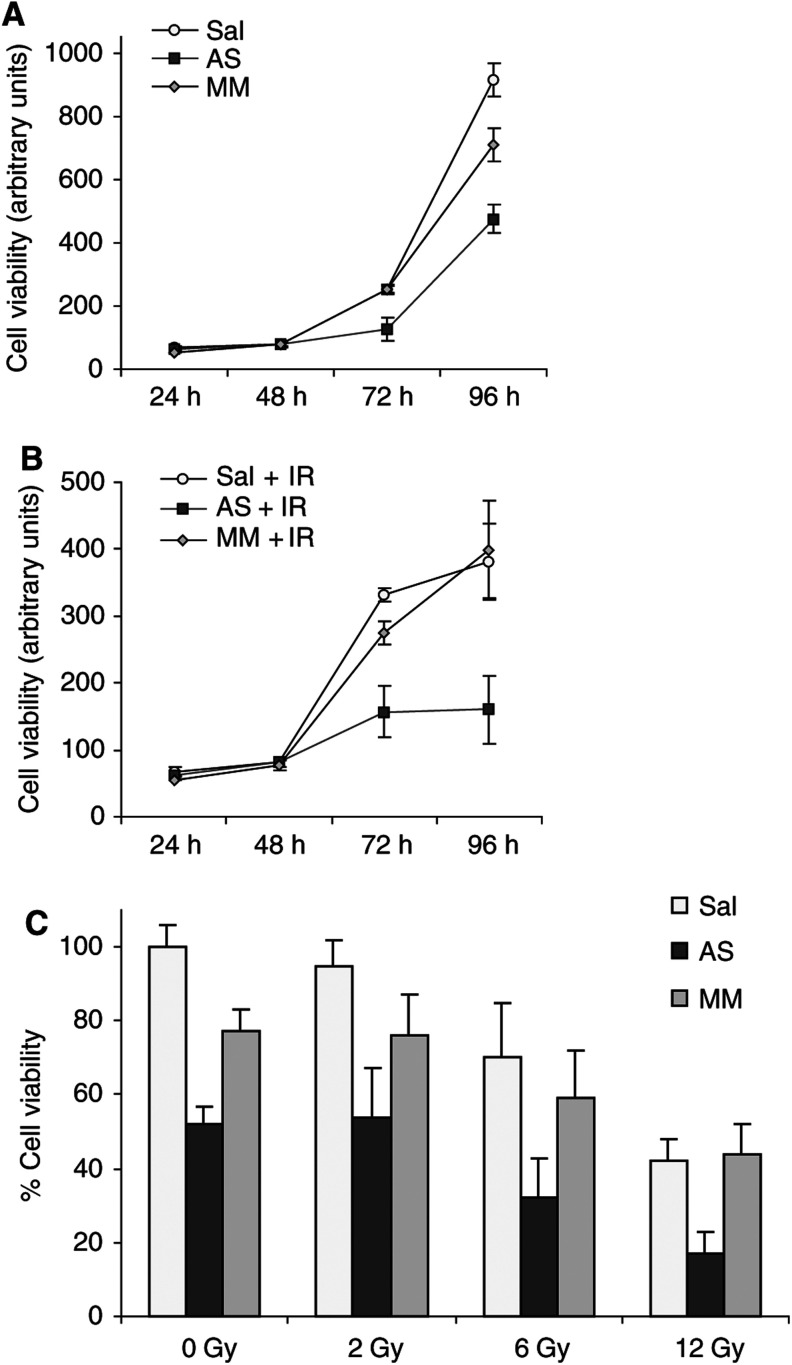
Bcl-x_L_ AS oligonucleotides radiosensitise human colon cancer cells. Time course of Caco-2 cells incubated with saline (Sal), antisense- (AS), or eight-base mismatch (MM) oligonucleotides at a concentration of 200 nM (**A**) alone, (**B**) in combination with IR (12 Gy 48 h after oligonucleotides). (**C**) Dose–response experiment of Caco-2 pretreated with saline (Sal), antisense (AS), or mismatch (MM) oligonucleotides at 200 nM and exposed to increasing IR doses (0–12 Gy). Cell viability was measured 96 h after oligonucleotide treatment by WST-1 assay. Representative data from four independent experiments are presented; bars=s.d.

We first determined cell viability after AS oligonucleotide mono-treatment in a time course experiment by the tetrazolium-based WST-1 assay (Figure 4A

). Bcl-x_L_ AS oligonucleotides alone significantly reduced the viability of Caco-2 cells compared to MM control or sham-treated cells beginning 72 h after incubation with oligonucleotides (Figure 4A; *P*<0.003). At 96 h, cell viability was reduced by one-third relative to the MM control (66% AS *vs* MM, s.d. ±13%; *P*<0.001). Cell viability of the MM oligonucleotide-treated cells did not differ from the saline control except at 96 h after oligonucleotide administration when a moderate inhibition of cell growth compared to saline treatment was observed (*P*<0.05).

For combination experiments, Caco-2 cells were exposed to IR 48 h after incubation with oligonucleotides. Starting from 72 h after AS oligonucleotide treatment, combinations of Bcl-x_L_ AS oligonucleotides and IR significantly reduced the viability of Caco-2 cells compared to controls (Figure 4B; all at least <0.005). In dose–response experiments, ISIS 16009 significantly sensitised human Caco-2 colon cancer cells to increasing IR doses of 2, 6 and 12 Gy by 30–60% relative to irradiated control cells (Figure 4C; *P*<0.05). MM oligonucleotide treatment combined with IR did not lead to results statistically significantly different from those obtained with irradiated saline groups at any dose or time point investigated.

**Figure 5:**
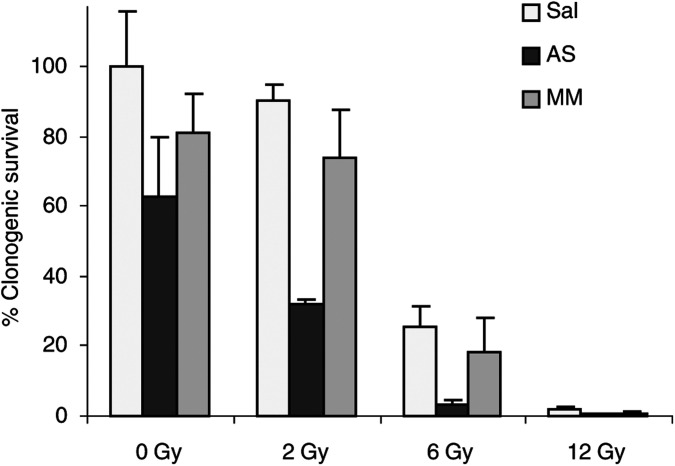
Bcl-x_L_ AS oligonucleotides decrease clonogenic survival of human colon cancer cells after ionising irradiation. Caco-2 cells were incubated with saline (Sal), antisense (AS), or eight-base mismatch (MM) oligonucleotides at a concentration of 200 nM in the presence of 10 *μ*g ml^−1^ lipofectin and irradiated 48 h later. Survival was assessed by colony-forming assay and expressed relative to solvent-treated cells. Columns represent mean percentages from three independent experiments; bars=s.d.

It is known that induction of apoptosis as well as tetrazolium-based short-term proliferation assays do not necessarily predict overall sensitivity of cancer cells to genotoxic treatment (Brown and Wouters, 1999). Especially for studies assessing the fraction of cells maintaining their reproductive integrity after IR, it is sensible to perform colony-forming assays. We therefore performed clonogenic assays of Caco-2 cells treated with Bcl-x_L_ AS oligonucleotides at increasing doses of IR (Figure 5

). Administration of ISIS 16009 alone resulted in a statistical nonsignificant trend towards reduced clonogenic survival compared to the MM control. However, the combination of Bcl-x_L_ AS oligonucleotides and IR at doses of 2 and 6 Gy significantly reduced colony formation in a dose-dependent manner by at least two-thrids compared to MM or saline pretreated cells (Figure 5; *P*<0.05). Again, MM oligonucleotide treatment combined with both IR doses did not differ statistically significantly from corresponding saline groups. At the highest radiation dose of 12 Gy, we observed no reliable colony formation in any treatment group.

We furthermore examined the chemosensitising effect obtained by the combination of Bcl-x_L_ AS oligonucleotides and cisplatin. Caco-2 cells treated with ISIS 16009 and cisplatin (50 *μ*M) revealed more than a 75% reduction in cell viability after 96 h compared to cisplatin-treated controls (78% AS+Cis *vs* Sal+Cis, s.d. ±5%; 77% AS+Cis *vs* MM+Cis, s.d. ±5%; both *P*<0.001; data not shown). Similar, clonogenic survival of Bcl-x_L_ AS oligonucleotide and cisplatin treated Caco-2 cells was significantly reduced by about 70% compared to the respective MM and saline controls (*P*<0.001; data not shown).

## DISCUSSION

Failure of cells to undergo apoptosis or programmed cell death may contribute to the treatment resistance of colon cancer (Kim *et al*, 1999). Decreasing the apoptotic threshold, mediated at least in part by the antiapoptotic Bcl-2 family member Bcl-x_L_, should lead to higher response rates of apoptosis-inducing treatment modalities (Maurer *et al*, 1998). In this study, we demonstrated a sensitisation of colorectal cancer cells to IR by specific downregulation of the long splicing variant of Bcl-x protein with Bcl-x_L_ AS oligonucleotides. This regulation lowered the apoptotic threshold and resulted in a pronounced inhibition of cell viability and clonogenic survival with a significant increase in IR-mediated apoptosis. In accordance with previous reports, the clonogenic survival assay was more sensitive than the tetrazolium-based proliferation assay, especially at higher radiation doses (Banasiak *et al*, 1999). This may be explained by the differences in the end points of both assays. The WST-1 tetrazolium assay (used for the time course experiments) scores the number of metabolically active cells, whereas the clonogenic assay is dependent on colony formation and therefore relies on cells that maintain their reproductive integrity (Banasiak *et al*, 1999). Thus, cells that have lost their reproductive potential immediately following treatment with ASO/irradiation or after a few cell divisions but which are still viable will still be scored by the WST-1 test, but not be recorded in the clonogenic assay.

High levels of antiapoptotic Bcl-2 family members are frequently found in tumours. Bcl-x_L_ and Bcl-2 both have the potential to block the process of apoptosis induced by the same stimuli (Huang *et al*, 1997). However, they may play nonredundant and distinct biological roles in cell survival and drug resistance depending on the type of tissue. There is growing evidence that among the antiapoptotic members of the Bcl-2 family, Bcl-x_L_ rather than Bcl-2 is a crucial factor responsible for the regulation of apoptotic cell death in colon cancer (Maurer *et al*, 1998). In more than 60% of all colon cancer, Bcl-x_L_ staining is more pronounced than in normal colon epithelium, whereas Bcl-2 expression was reported to be too low for detection by Northern blotting (Krajewska *et al*, 1996; Maurer *et al*, 1998). There is a significant correlation between the chemosensitivity of this malignancy and the Bcl-x_L_ to Bax ratio, which is not observed to the same extent in the Bcl-2 to Bax ratio (Nita *et al*, 1998).

A screening approach using micromolar concentrations of four different Bcl-x_L_ AS oligonucleotides led us to select ISIS 16009 as the AS oligonucleotide reducing Bcl-x_L_ expression most potently. Using lipofectin as a cationic uptake enhancer allowed us to reduce ISIS 16009 AS oligonucleotides concentrations to the nanomolar range that minimises nonantisense oligonucleotide effects reported to occur at micromolar concentrations (Stein, 1995). Notably, ISIS 16009 Bcl-x_L_ AS oligonucleotides did not reduce the alternative, short splicing proapoptotic variant of the Bcl-x gene nor did they shift the splicing pattern of Bcl-x pre-mRNA from Bcl-x_L_ to Bcl-x_S_ (Mercatante *et al*, 2002). This finding further supports a specific antisense mechanism of action for the Bcl-x_L_ AS oligonucleotide used in this study.

Cellular susceptibility to apoptosis is thought to be determined by the ratio of pro- and antiapoptotic Bcl-2 family members rather than the total amounts present in a given cell (Tsujimoto and Shimizu, 2000). In this study, downregulation of the Bcl-x_L_ protein product by about 50% compared to MM- or saline control-sensitised Caco-2 colorectal cancer cells to IR or cisplatin. Since it was not necessary to block completely Bcl-x_L_ expression to observe the effects demonstrated, our findings support the hypothesis of a critical balance between pro- and antiapoptotic factors in the tightly regulated process of apoptosis. This is of special interest since it is known that proapoptotic Bax mRNA is overexpressed in 75% of colorectal cancer specimen (Maurer *et al*, 1998). In the relative absence of its heterodimer partner Bcl-x_L_ due to AS oligonucleotide treatment, Bax should preferentially form homodimers resulting in facilitated programmed cell death upon apoptotic stimulation.

The development of antisense technology represents a promising strategy to improve conventional therapy outcomes. For colon cancer, several apoptosis-related targets for AS oligonucleotide approaches have already been tested. Treatment with EGFR AS oligonucleotides showed an inhibition of human colon cancer cell growth with potentiation of inhibitory cell growth effects in combination with cytotoxic drugs (Ciardiello *et al*, 2001). p21 AS oligonucleotides sensitised colon cancer cells *in vivo* by downregulation of IR induced p21 expression and increased apoptotic cell death (Tian *et al*, 2000). Bispecific AS oligonucleotides targeting Bcl-x_L_ and Bcl-2 have been shown to reduce colon cancer cell growth *in vitro* and *in vivo* (Gautschi *et al*, 2001). Combination strategies with chemotherapy, a concept even more attractive in theory, have not been addressed in this study.

Bcl-x_L_ AS oligonucleotides in combination with the cytostatic agent 5-fluorouracil have been reported recently to increase apoptosis and reduce cell growth by 40% in colon cancer cells (Nita *et al*, 2000). In our study, using a different Bcl-x_L_ AS oligonucleotide sequence in a different colon cancer cell line, the chemosensitisation approach was successfully extended to a more than 70% reduction of cell viability in combination with the cytotoxic chemotherapeutic agent cisplatin. Single-agent Bcl-x_L_ AS oligonucleotide treatment had effects similar to those reported in the study mentioned above. However, considering possible therapeutic applications, systemic administration of myelosuppressive chemotherapy in combination with Bcl-x_L_ AS oligonucleotides may lead to harmful side effects. Among the antiapoptotic Bcl-2 family members, Bcl-x_L_ rather than Bcl-2 is presumed to be a key player in the survival of haematopoietic cell lineages, developing megakaryocytes and for the lifespan of mature platelets (Sanz *et al*, 2001). Even though clinical data for Bcl-x_L_ AS oligonucleotides are not yet available, it will be prudent to monitor closely the patients treated with combinations of myelosuppressive chemotherapeutics such as 5-fluorouracil or cisplatin for haematological side effects. As an indirect line of support for this concern, thrombocytopenia as dose-limiting toxicity as well as transient leucopenia have been observed in the first clinical trial combining a mild myelosuppressive standard chemotherapeutic regimen with Bcl-2 AS oligonucleotides in melanoma (Jansen *et al*, 2000).

It appears reasonable to speculate that combining the systemic administration of Bcl-x_L_ AS oligonucleotides with a localised treatment approach such as IR restricted to the tumour site could circumvent or at least minimise anticipated dose-limiting haematological side effects without negative impact on its sensitisation effect on tumor cells.

In this study, we report that Bcl-x_L_ AS oligonucleotides are capable of sensitising colon cancer cells to IR, one of the most commonly used treatment strategies for localised colorectal cancer in an adjuvant setting. Cell viability and clonogenic survival in Bcl-x_L_ AS oligonucleotides pretreated colon cancer cells was blocked by about 60% compared to irradiated control cells. These findings underline the role of Bcl-x_L_ protein as a resistance factor in colon cancer and as an attractive target for therapeutic concepts capable of specifically modulating protein expression such as in AS oligonucleotides strategies. Certainly, these promising first data deserve further evaluation and need to be confirmed in preclinical animal models. However, given the feasibility of AS oligonucleotide administration reported from first clinical trials (Jansen and Zangemeister-Wittke, 2002), the results reported here may provide the basis for the use of Bcl-x_L_ AS oligonucleotides as a rational radiosensitising strategy to help improve treatment outcome in colon cancer patients.
